# Psychological, pharmacological, and combined smoking cessation interventions for smokers with current depression: A systematic review and meta-analysis

**DOI:** 10.1371/journal.pone.0188849

**Published:** 2017-12-05

**Authors:** Roberto Secades-Villa, Alba González-Roz, Ángel García-Pérez, Elisardo Becoña

**Affiliations:** 1 Department of Psychology, University of Oviedo, Oviedo, Spain; 2 Department of Clinical Psychology and Psychobiology, University of Santiago de Compostela, Santiago de Compostela, Spain; University of Bern, SWITZERLAND

## Abstract

We conducted a systematic literature review and meta-analysis (ID: CRD42016051017) of smoking cessation interventions for patients with current depression. We examined the effectiveness of smoking cessation treatments in improving abstinence rates and depressive symptoms. The following electronic databases were used for potentially eligible studies: PUBMED, PSYCINFO, DIALNET and WEB OF KNOWLEDGE. The search terms used were: smoking cessation, depressive disorder, depression, mood, depressive, depressed, smoking, smokers, nicotine, nicotine dependence, and tobacco cigarette smoking. The methodological quality of included studies was assessed using the Effective Public Health Practice Project Quality assessment tool (EPHPP). Of the 6,584 studies identified, 20 were eligible and included in the review. Trial designs of studies were 16 randomized controlled trials and 4 secondary studies. Studies included three types of intervention: psychological (6/30%), pharmacological (6/30%) or combined (8/40%). Four trials comprised special populations of smokers. Four studies received a strong methodological quality, 7 were scored as moderate and 9 studies received a weak methodological rating. Analyses of effectiveness showed that smoking cessation interventions appear to increase short-term and long-term smoking abstinence in individuals with current depression. Subgroup analyses revealed stronger effects among studies that provided pharmacological treatments than in studies using psychological treatments. However, the evidence is weak due to the small number of studies. Smoking abstinence appears to be associated with an improvement in depressive symptoms. Heterogeneity in protocols in similar types of treatment also prevent firm conclusions being drawn on the effectiveness of any particular treatment model to optimally manage abstinence among depressed smokers. Further research is required to strengthen the evidence base.

## Introduction

Tobacco smoking is one of the main risk factors for many chronic illnesses and the leading preventable cause of morbidity and premature death worldwide [1]. Smoking is particularly prevalent in the portion of the population suffering from depression [2]. People with depression are about twice as likely to be smokers than are individuals who are not depressed [3]. Furthermore, smokers with depression are more likely to meet criteria for nicotine dependence, more likely to suffer from negative mood changes after nicotine withdrawal, and are less likely to succeed at cessation attempts compared to those without depression [4].

The association between the two conditions is likely bidirectional, with smoking resulting in mood changes and smoking being a compensatory behavior to alleviate symptoms of depression [5, 6].

Despite the fact that standard smoking cessation interventions may be less effective for people with depression than evidenced in the general population, previous studies have found several promising interventions, including behavioral counseling, behavioral mood management, or nicotine replacement therapy (NRT) [7, 8].

Of further concern is the fact that tobacco cessation will compromise depression prognosis; however, a number of studies have reported improvements in the symptoms of depression following smoking cessation [9–11]. Nevertheless, the evidence is still scarce and more research is needed.

Despite the evidence regarding smoking rates in people with depression, little is known about smoking treatment options for this population and more research is needed to identify successful interventions. Systematic reviews and meta-analyses of smoking cessation interventions in people with depression have been done, but the issue of managing co-occurring conditions has not been examined closely. These reviews included patients who had a history of depression but not current depression [7, 8, 12–14], patients who did not meet criteria for depression [7, 8], or studies that did not assess the effects of the intervention and smoking status on depressive symptoms [7, 8, 12, 13]. No reviews of the effectiveness of smoking cessation interventions for patients solely with current depression exist. This is important if we consider that patients with a history of depression may respond differently to smoking cessation treatment than patients with current depression. Consequently, very little is known about the effectiveness of smoking cessation treatments and how current depression affects smoking cessation in this population [13].

In order to address these gaps in knowledge, the primary aim of this review and meta-analysis was to evaluate the effectiveness of smoking cessation interventions for patients with current depression. The secondary aim was to evaluate the impact of smoking cessation treatments on the symptoms of depression. Finally, the quality of the included studies was also evaluated.

As many mental health services do not offer smoking cessation treatment [15] it is hoped that the findings will provide clearer direction on how to incorporate smoking cessation into depression interventions.

## Method

For the purposes of this study, a protocol was designed and registered in the International Prospective Register of Systematic Reviews, PROSPERO (ID: CRD42016051017). The systematic review and meta-analysis was conducted following the Preferred Reporting Items for Systematic Reviews (PRISMA statement)(S1 Table) [16]. The study was supported by the Spanish Ministry of Economy and Competitiveness, the European Regional Development Fund (Grant PSI2015-64371-P. MINECO/FEDER), and by the Predoctoral Grants, BES-2016-076663, from the Spanish Ministry of Economy and Competitiveness (MINECO), and FPU15/04327, from the Spanish Ministry of Education, Culture and Sport. The funding sources played no role in the study design, data collection, analysis or interpretation of the results.

### Literature search procedure

**Fig 1:**
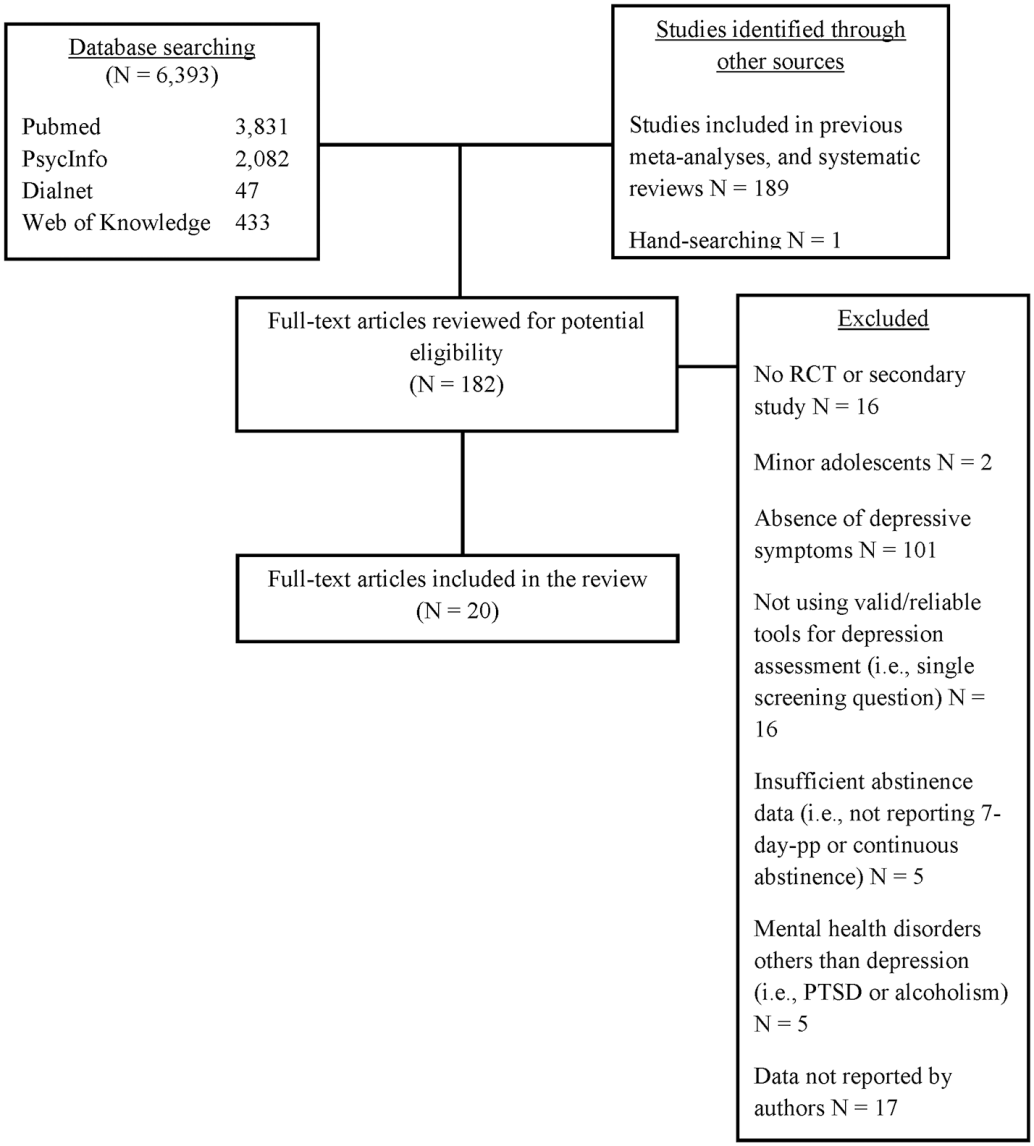
Literature search procedure.

A comprehensive literature review search, up to September 2017 was carried out (see Fig 1). No restriction on the year of publication was considered. Authors first conducted a search of studies included in both systematic reviews and meta-analyses focused on smoking and depression. Additionally, the following electronic databases were used for potentially eligible studies: PUBMED, PSYCINFO, DIALNET and WEB OF KNOWLEDGE. For the purpose of this review, the search terms used were: smoking cessation, depressive disorder, depression, mood, depressive, depressed, smoking, smokers, nicotine, nicotine dependence, and tobacco cigarette smoking. Full-text versions of articles identified through the literature search (n = 183) were further evaluated for eligibility in the systematic review by two independent reviewers. When discrepancies occurred, a decision regarding whether or not to include an article was reached by a third independent reviewer.

### Eligibility and exclusion criteria

Both randomized clinical trials and secondary studies were considered for inclusion in this review if they met the following criteria: 1) they provided a smoking cessation treatment for smoking cessation; 2) they comprised samples of adult smokers with current major depression or depressive symptoms; 3) they used reliable and valid tools for depression assessment (i.e., structured or semi-structured interviews based on DSM criteria or multi-item scales); and 4) they reported a measure of smoking cessation (point prevalence, prolonged, or continuous abstinence). Studies including individuals with mental health disorders others than depression (e.g., alcoholism, post-traumatic stress disorder) were discarded.

### Data extraction

Two trained researchers abstracted data from the included studies and checked the data of the third researcher. We abstracted information about: participants (sample size, percent female, mean age, mean number of cigarettes per day and mean depressive symptoms), method (setting, depression assessment and study design), interventions (description and type of interventions) and outcomes (definition of abstinence, length of follow-up and biochemical validation). When authors did not report sufficient data, we asked the first author for additional data not supplied in the full text (e.g., mean of depressive symptoms for the total sample).

### Narrative synthesis

In the narrative syntheses, we qualitatively reviewed study findings in the context of study design and characteristics, including: participants’ characteristics (i.e., age, number of cigarettes smoked), measurement of depression, biochemical validation of smoking abstinence, follow-up periods, settings and treatment conditions.

### Data analysis

Meta-analysis was conducted by Comprehensive Meta-Analysis [17]. Results for abstinence were expressed as risk ratios (RRs) with 95% confidence intervals (CIs) for each study. An RR greater than 1 favored the active group (i.e., individuals receiving the treatment under study) for improved abstinence with regard to the comparison condition (i.e., individuals not receiving the treatment under evaluation). For studies with multiple intervention conditions, intervention groups were collapsed and compared with the comparison group [18]. We examined heterogeneity of pooled trials with Cochran´s Q test; *p* values less than 0.10 are considered significant [19]. I^2^ statistic describes the grade of variability; values around 25%, 50% and 75% can be interpreted as a low, medium, and high heterogeneity respectively [20]. We performed subgroup analyses separately for types of intervention (psychological and pharmacotherapy) and length of follow-up (short-term abstinence (≤ 3 months) and long-term abstinence (6 or 12 months). Whenever possible, 7-day point prevalence was used as the criterion of abstinence.

In addition to frequency and descriptive statistics, we reported all statistically significant and non-significant outcomes by study. Additionally, findings were calculated for each treatment arm. Due to the heterogeneity in terms of designs, follow-up periods, and treatment characteristics, meta-analysis of depression was not performed and the study findings were synthesized narratively.

### Methodological quality assessment

The methodological quality assessment of each of the included studies was conducted by two independent reviewers using the Effective Public Health Practice Project Quality assessment tool (EPHPP) [21]. This tool is widely used to evaluate a variety of intervention study designs, such as randomized clinical trials, and it has been judged suitable to be used in systematic reviews [22]. It comprises six domains: (1) selection bias; (2) study design; (3) confounders; (4) blinding; (5) data collection methods and; (6) withdrawals/drop-outs. Based on its total score, each study is assigned a global quality rating of weak, (1.00–1.50), moderate (1.51–2.50), or strong (2.51–3.00). Consensus between reviewers was obtained.

## Results

A total of 6,584 articles were identified through the literature search and individually examined (Fig 1). Based on the title and the abstract, a full text screen of 183 articles was performed. Of the reviewed articles twenty (11.2%) studies, published between 1997 and 2017, met the inclusion criteria and therefore were included in this review. Table 1 shows a summary of characteristics for the revised studies (sixteen RCTs and four secondary studies). For study data, see S1 Dataset.

**Table 1:** Study characteristics.

Author (year)	Sample size (% female)	AgeMean± *SD*	Cigarettes Mean±*SD*	Depression assessment	Depression		Setting	Conditions
					Diagnosis (%)	Depressive Symptoms (mean)		
Anthenelli et al. (2013)	525 (62.7%)	46.27±10.85	21.70±8.12	MADRS SCID	-	7.76	Academic clinical trial centers and smoking cessation clinics	Varenicline vs Placebo (both with counseling)
Bernard et al. (2015)	70 (58.6%)	48.45±10.45	21.45±8.90	HADS-D MINI	MDD (7.1%)	10.45	Montpellier University Hospital	Exercise and Counseling vs Health Education Control (both with NRT or Varenicline)
Catley et al. (2003)	498 (60%)	42.95±10.40	19.80±10.20	SDS	-	0.16 (35.5% probable depression)	Inner-city hospital	Culturally sensitive material (guide + video) vs Standard material (guide + video) (both with NRT)
Cinciripini et al. (2010)	257 (100%)	25.00±5.90	16.30±9.00	CES-DSCID	MDD (23.3%)	18.8	Clinic	CBASP vs HW (both with counseling)
Evins et al. (2008)	199 (49%)	43.00±11.00	25.00±11.00	HAM-D SCID	MDD (34.2%)	10.6	Hospital	Bupropion vs Placebo (both with CBT + NRT)
Hall et al. (2006)	322 (69.6%)	41.84±12.60	15.55±10.15	PRIME-MD BDI-II	MDD (83.2%) MDD-R (52.2%)	21.00	Mental health outpatient clinics	Staged Care Intervention (NRT and bupropion under request) vs Brief Contact Control
Hayes et al. (2010)	237 (53.6%)	56.13±14.08	21.11±14.00	CES-D	-	21.21	Home visits	Standard Care vs Motivational Enhancement
Japuntich et al. (2007)	71^a^ (64.8%)	41.25±11.55	28.75±10.74	PRIME-MD	MDD (100%)	-	Clinic	Motivational interviewing vs CBT (both with NRT and brief individual counselling)
Kinnunen et al. (2008)	196^a^ (56.1%)	38.5±11.3	23.5±11.1	CES-D	-	24.7	Harvard School of Dental Medicine	Nicotine gum (NRT) vs Placebo gum (both with brief behavioral counselling)
Minami et al. (2015)	45^a^ (48.9%)	46.1±11.5	-	CES-D SCID	-	16.07	Medical context	ST-Fluoxetine vs SEQ-Fluoxetine vs NRT (all with counseling + NRT)
Muñoz et al. (1997)	136 (38.2%)	35.3	14.1±8.2	CES-DMV-DIS^21^ depression section	MDD (39.0%)	21.3	Self-help	Guide vs Guide + MM^22^ (both with two conditions: delayed and immediate)
Muñoz et al. (2006, study 3)	280 (67.9%)	38.4±10.8	20.3±9.7	CES-D MDE screener	MDD (11.4%)	16.2	Internet based self-help	Guide+ ITEMs vs Guide + ITEMs + MM (both suggested using NRT)
Muñoz et al. (2006, study 4)	288 (41.3%)	35.0±9.5	22.8±10.2	CES-DMDE screener	MDD (16.7%)	15.9	Internet based self-help	Guide + ITEMs vs Guide + ITEMs + MM (both suggested using NRT)
Muñoz et al. (2009)	1,000 (45%)	37.9±11.3	19.8±10.1	CES-D MDE screener	MDD (12.9%)	16.0	Internet based self-help	Guide vs Guía+ITEMs vs Guide+ITEMs+MM vs Guide+ITEMS+MM+VG (both suggested using NRT)
Patten et al. (2017)	30 (100%)	37.5±10.5	-	PHQ-9	-	11.7	Clinic	Exercise vs Health Education (both with counselling and NRT)
Schnoll et al. (2010)	55^a^ (63.63%)	51.67	17.52	CES-D	-	-	Not described	Bupropion vs Placebo (both with counseling and NRT)
Thorsteinsson et al. (2001)	38 (47.4%)	46.26±9.6	28	SCID HAM-D BDI	MDD (100%)	18.1 20.9	Not described	NRT vs Placebo (both with counseling)
van der Meer et al. (2010)	485 (76.5%)	43.75±10.05	21.60±9.30	CES-D	-	16.65	Dutch national quitline	Mood Management intervention vs Control (both with counselling and may include NRT, bupropion or nortriptyline)
Vickers et al. (2009)	60 (100%)	41.35±11.95	20.8±7.55	CES-D HAM-D	-	31.1 14.1	Clinic	Exercise counseling vs Health Education (both with counselling and NRT)
Ward et al. (2013)	269 (21.55%)	40±11.4	27.74±12.69	CES-D	-	18.04	Primary care clinics	Nicotine patch vs Placebo (both with behavioral cessation counseling and brief telephone support)

MADRS = Montgomery–Åsberg Depression Rating Scale; SCID = Structured Clinical Interview for DSM Disorders; HADS-D = Hospital Anxiety and Depression Scale-Depression; MINI = Mini International Neuropsychiatric Interview; MDD = Major depressive disorder; NRT = Nicotine Replacement Therapy; SDS = Short Depression Screen; CES-D = Center for Epidemiological Studies-Depression; CBASP = Cognitive Behavioral Analysis System of Psychotherapy; HW: Health and Wellness Education; HAM-D = Hamilton Rating Scale for Depression; CBT = Cognitive behavioral therapy; PRIME-MD = Primary Care Evaluation of Mental Disorders; BDI-II = Beck Depression Inventory; MDD-R = Recurrent major depressive disorder; ST-Fluoxetine = Standard fluoxetine treatment; SEQ-Fluoxetine = Sequential fluoxetine treatment, starting 8 weeks since pre-quit; MV-DIS depression section = Modified version of the depression section of the diagnostic interview schedule (DIS); MM = Mood Management; MDE screener = The major depressive episode screener; ITEMs = Individually timed educational messages; VG = Virtual group.

^a^Only depressed smokers.

### Participants’ characteristics

Participants were adult smokers (aged 18 or more) with depressive symptoms and/or a diagnosis of a current major depressive disorder. Five of nineteen studies included special populations: individuals with cancer, medically ill individuals, pregnant women, and smokers with low income levels. The total number of patients was 5,061. The sample sizes of the included studies ranged from 30 to 1,000 with a mean of 253.1 (SD = 235.7). The mean age of the total sample was 40.9. Half of the participants in the reviewed studies were females (58.0%) and the average number of cigarettes smoked per day in the baseline was 21.

### Study and treatments characteristics

Most of the studies (15/75%) used a biochemical validation (carbon monoxide or cotinine) to assess smoking abstinence. The criterion of abstinence most common was point prevalence (18/90%), followed by continuous abstinence rate (5/25%), and prolonged abstinence (3/15%).

Two of the reviewed studies (10%) only included abstinence at the end of treatment. Eight studies (40%) reported a minimum follow-up period of 6 months and 10 (50%) collected data at 12 months or more.

Studies included in this review evaluated three types of intervention: psychological (6/30%), pharmacological (6/30%), or combined (8/40%). Thirteen studies (65%) assessed the effect of psychological interventions for smoking cessation, exclusively (6/30%) or combined with pharmacotherapy (7/35%). Cognitive behavioral therapy (CBT) was included in three studies [23–25]. Two studies assessed the effect of motivational interview (MI) techniques [25, 26]. Self-help materials were provided in five trials [27–30]. Finally, three studies [31–33] included exercise interventions.

Ten trials (50% of the total) evaluated a psychological treatment with a mood management component, solely [23, 27, 29] or combined with pharmacotherapy [24, 28, 30–33]. Six of the aforementioned studies included behavioral activation (BA) techniques such as increasing pleasant activities and daily mood monitoring [24, 27–30].

Seven trials (35%) assessed the effect of pharmacotherapy for smoking cessation, solely [34–39] or combined with psychological treatment [40].

Two studies assessed the effect of NRT exclusively [35, 38]. Ten studies used NRT in combination with other interventions. Of them, six combined NRT with psychological treatments [24, 25, 31–33, 41] and two with anti-depressants (fluoxetine and bupropion, respectively) [36, 37]. The remaining study combined NRT with both anti-depressants and psychological treatments [30, 40].

All the studies providing anti-depressants added NRT [24, 30, 36, 37, 40, 42]. Of them, only two studies [36, 37] did not include psychological treatments.

There was one study testing the effect of varenicline exclusively [34]. Bernard, Ninot [31] combined varenicline with psychological treatment and NRT.

### Meta-analyses: Smoking outcomes

**Fig 2:**
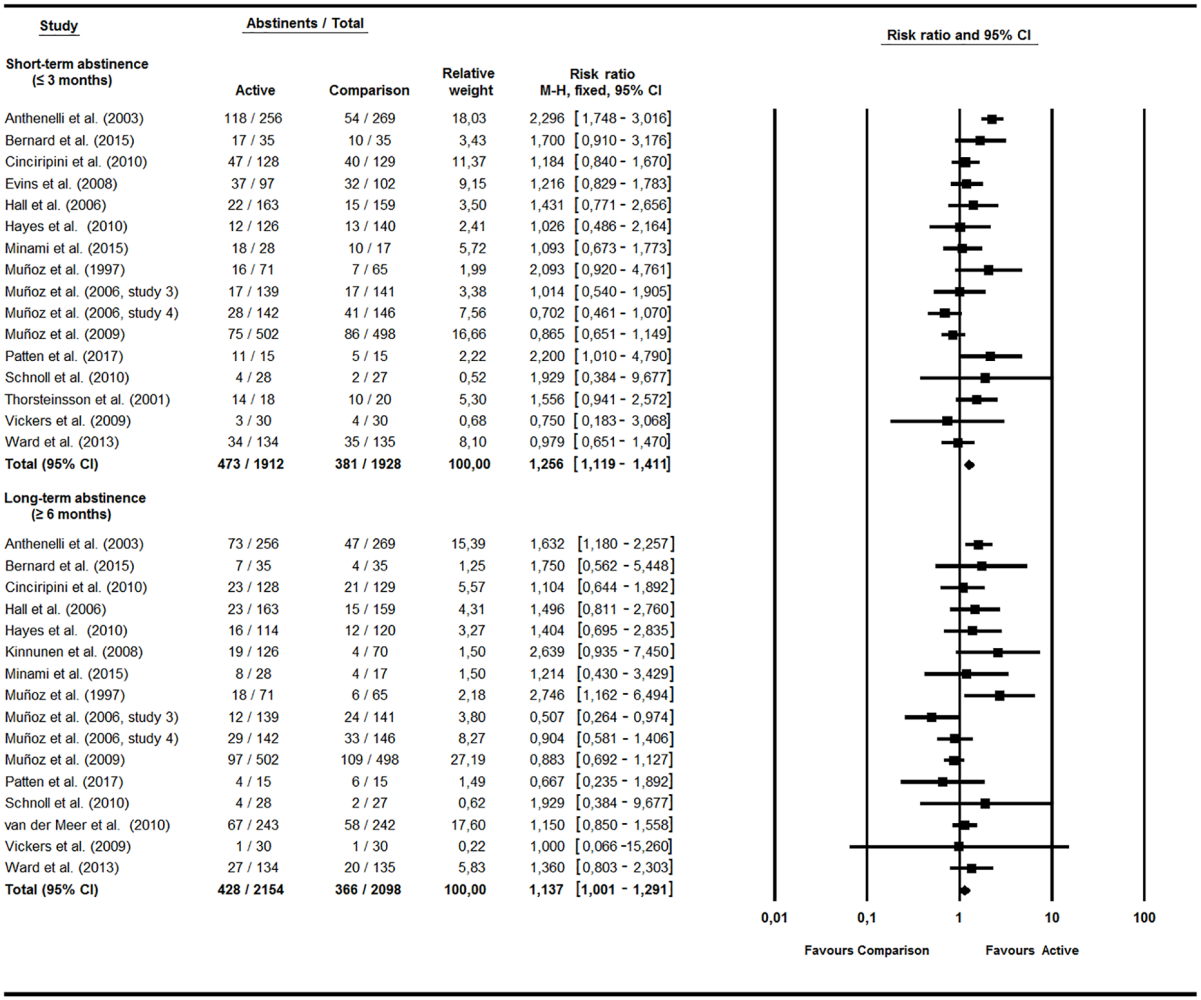
Psychological, pharmacological, or combined interventions.

Meta-analyses (Fig 2) revealed higher abstinence rates in the intervention relative to the comparison condition in both the short (RR = 1.26, 95% CI = 1.12–1.41, *p* < .001, Q(15) = 41.39, *p* < .001, I^2^ = 63.76%) and long-term (RR = 1.14, 95% CI = 1.01–1.29, *p* = .048, Q(15) = 25.97, *p* = .038, I^2^ = 42.24%). Abstinence rates in the active and comparison conditions at short-term were 27.74% and 19.76%, respectively. At long-term, 19.87% and 17.45% of patients remained abstinent.

Two additional studies performed secondary analyses and hence could not be included in the meta-analysis. Catley, Ahluwalia [41] used culturally sensitive self-help material, including a guide and a video for depressed smokers. Despite these authors reporting abstinence rates at 6-month follow-up among the overall sample (25%), they do not account for treatment condition. Japuntich, Smith [25] analyzed the relationship between depression and smoking after receiving MI or CBT for quitting. Again, although they report a 6-month follow-up abstinence of 21%, these authors do not account for treatment condition, so conclusions on the effect for each treatment cannot be established.

#### Effects of psychological treatments on smoking abstinence

**Fig 3:**
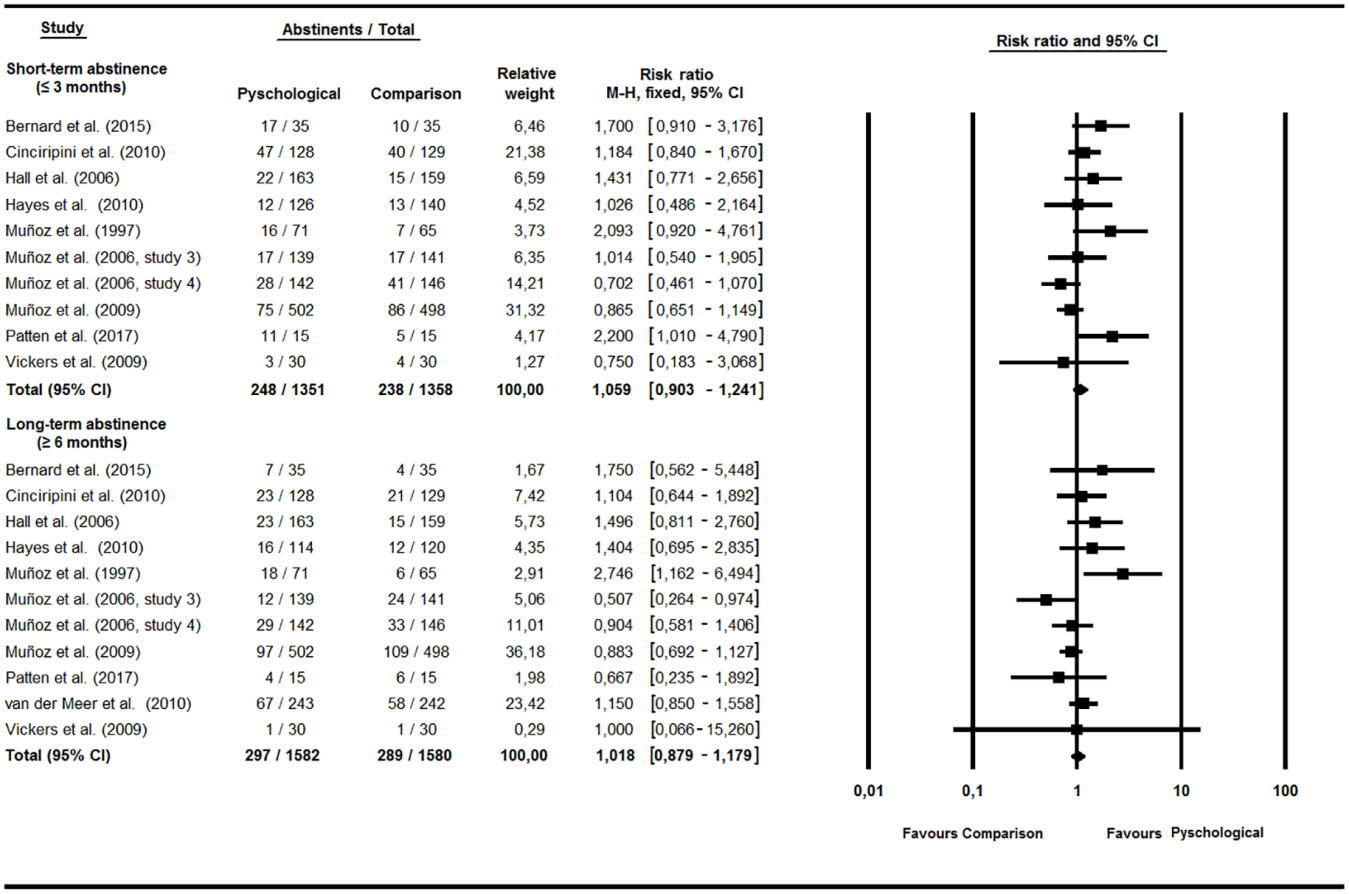
Psychological interventions.

Meta-analysis found a positive effect, although not significant, for psychological treatments against a comparison condition at both short (RR = 1.06, 95% CI = 0.90–1.24, *p* = .48, Q(9) = 15.41, *p* = .08, I^2^ = 41.58%) and long-term follow-up (RR = 1.02, 95% CI = 0.88–1.18, *p* = .809, Q(10) = 15.62, *p* = .11, I^2^ = 35.99%) (Fig 3). Results showed the strongest effects for the simultaneous BA treatment of depression and tobacco dependence at long-term (RR = 2.75, 95% CI = 1.16–6.49, *p* = .02), with a 25.4% abstinence.

#### Effects of pharmacological treatments on smoking abstinence

**Fig 4:**
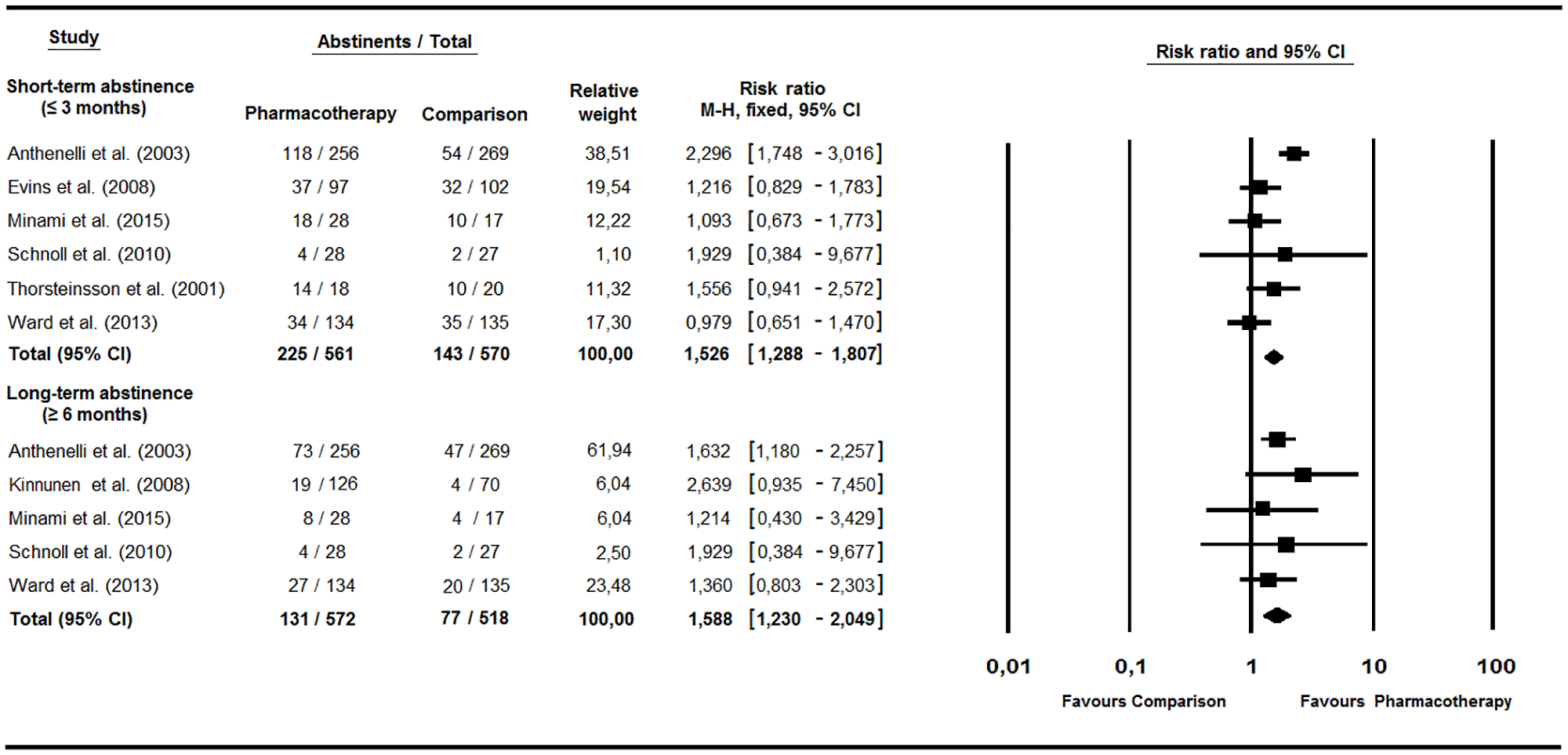
Pharmacological interventions.

Analysis showed a favorable effect for pharmacotherapy at three or fewer month follow-up (RR = 1.53, 95% CI = 1.29–1.81, *p* < .001, Q(5) = 16.46, *p* = .006, I^2^ = 69.63%). This effect remained significant in the long-term (RR = 1.59, 95% CI = 1.23–2.05, *p* < .001, Q(4) = 1.59, *p* = .81, I^2^ = 0%) (Fig 4). Subgroup analysis yielded the strongest effects for varenicline in each of the time-frame assessments (RR = 2.30 and 1.63, respectively).

### Systematic review: Smoking outcomes

Table 2 shows a summary of findings for the effect of smoking cessation treatments on tobacco abstinence and depression outcomes. Analyses were also performed separately for psychological treatments and pharmacotherapy.

**Table 2:** Effect of smoking cessation treatments on abstinence rates and depressive symptoms.

Author (year)	Smoking outcomes	Depression outcomes
Anthenelli et al. (2013)	CA weeks 9–12: varenicline: 35.9% vs placebo: 15.6%, *p* ≤ .001 weeks 9–24: varenicline: 25% vs placebo: 12.3%, *p* ≤ .001 weeks 9–52: varenicline: 20.3% vs placebo: 10.4%, *p* ≤ .001 PP week 12: varenicline: 46.1% vs placebo: 20.1%, *p* ≤ .001 week 24: varenicline: 31.3% vs placebo: 18.2%, *p* ≤ .001 week 52: varenicline: 28.5% vs placebo: 17.5%, *p* = .002	Not reported
Bernard et al. (2015)	CA week 8 (EOT): exercise: 57.1% vs control: 37.1%, *p* = .09 CA 12 weeks: exercise: 48.6% vs health education: 28.6%, *p* = .08 CA 24 weeks: exercise: 34.3% vs health education: 22.9%, *p* = .28 CA 52 weeks: exercise: 20% vs health education: 11.4%, *p* = .32	HADS-D week 8: exercise vs control (*M* = 5.92; *SD* = 4.41 vs *M* = 5.36; *SD* = 3.38) HADS-D 12 weeks: exercise vs control (*M* = 5.25; *SD* = 4.87 vs *M* = 5.63; *SD* = 3.51) HADS-D 24 weeks: exercise vs control (*M* = 6.50; *SD* = 4.96 vs *M* = 6.66; *SD* = 4.56) HADS-D 52 weeks: exercise vs control (*M* = 3.87; *SD* = 2.89 vs *M* = 4.83; *SD* = 3.78)
Catley et al. (2003)	PP week 4: 37% PP at 6 month follow-up: 25%	% of participants above the cutoff in SDS: 4 week: 31.9% 6 months: 35.2%
Cinciripini et al. (2010)	CA EOT (visit 10): not reported 3 months after EOT: CBASP: 23.4% vs HW: 21%, *p* ≥ .05 6 months after EOT: CBASP: 11.1% vs HW: 8.5%, *p* ≥ .05 3 months postpartum: CBASP: 11.7% vs HW: 10.9%, *p* ≥ .05 6 months postpartum: CBASP: 3.1% vs HW: 1.2%, *p* ≥ .05 PP EOT (visit 10): CBASP: 45.3% vs HW: 39.2%, *p* ≥ .05 3 months after EOT: CBASP: 36.7% vs HW: 31.0%, *p* ≥ .05 6 months after EOT: CBASP: 18.0% vs HW: 16.3%, *p* ≥ .05 3 months postpartum: CBASP: 18.8% vs HW: 17.8%, *p* ≥.05 6 months postpartum: CBASP: 7% vs HW: 9.3%, *p* ≥.05 PA EOT (visit 10): not reported 3 months after EOT: CBASP: 31.3% vs HW: 27.1%, *p* ≥ .05 6 months after EOT: CBASP: 14.1% vs HW: 14.7%, *p* ≥ .05 3 months postpartum: CBASP: 16.4% vs HW: 18.6%, *p* ≥ .05 6 months postpartum: CBASP: 7.8% vs HW: 6.2%, *p* ≥ .05	Significant effect of treatment condition, *p* = .04, time, *p* ≤ .001, treatment group by time interaction, *p* ≤ .003 Raw scores I^:^ *M* = 5.53; SD = 2.87 Raw scores II^:^ *M* = 13.11; SD = 2.06 Raw scores III^:^ *M* = 21.16; SD = 3.44 Raw scores IV^:^ *M* = 35.08; SD = 5.07
Evins et al. (2008)	PP end of treatment: 34% (total sample): Bupropion + NRT + CBT = 36% vs placebo + NRT + CBT = 31%, *p* = NA PP end of treatment among current UDD vs past UDD: 32% vs. 35%, *p* = NA PP end of treatment among current UDD: Bupropion + NRT + CBT = 33% vs placebo + NRT + CBT = 31%, *p* = NA PP end of treatment among past UDD: Bupropion + NRT + CBT = 39% vs placebo + NRT + CBT = 32%, *p* = NA	HAM-D abstinents vs smokers at EOT: *M* = 9.80; SD = 6.33 vs *M* = 10.94; SD = 6.30, *p* = .278
Hall et al. (2006)	PP at 3 months: stage care: 13.5% vs brief contact: 9.43%, *p* = NA PP at 6 months: stage care: 14.11% vs brief contact: 15.73%, *p* = NA PP at 12 months: stage care: 14.11% vs brief contact: 9.43%, *p* = NA PP at 18 months: stage care: 18.40% vs brief contact: 13.21%, *p* = NA	Data not reported
Hayes et al. (2010)	PP EOT: SC: 7.9% vs ME: 8.8%, *p* = NA 2 months: SC: 8.8% vs ME: 9.3%, *p* = NA 6 months: SC: 10.1% vs ME: 11.2%, *p* = NA 12 months: SC: 8.5% vs ME: 12.6%, *p* = NA CA EOT: SC: 0.7% vs ME: 1.6%, *p* = NA 2 months: SC: 2.2 vs ME: 5.9%, *p* = NA 6 months: SC: 3.1% vs ME: 5.2%, *p* = NA 12 months: SC: 4.2% vs ME: 8.7%, *p* = NA	Data not reported
Japuntich et al. (2007)	% of CD abstinents 1 week: 4.2% 6 weeks: 36.6% 3 months: 31% 6 months: 21.1% % of PDO abstinents 1 week: 25% 6 weeks: 38% 3 months: 28.3% 6 months: 18.5% % of NHD abstinents 1 week: 64.2% 6 weeks: 37% 3 months: 34.8% 6 months: 19.8%	Data not reported
Kinnunen et al. (2008)	CA at 12 months: placebo vs NRT: depressed: 5.7% vs 15.1%, *p* = 0.5; non-depressed: 9.77% vs 20.1%, *p* = .009	Data not reported
Minami et al. (2015)	PP 2 weeks after quitting^a^: 60%; SEQ-Fluoxetine: 60% vs ST-Fluoxetine: 53.9% vs TNP: 64.7%, *p* = NA PP at 4 weeks after quitting^a^: 55.6%; SEQ-Fluoxetine: 66.7% vs ST—Fluoxetine: 38.5% vs TNP: 58.8%, *p* = NA PP at 8 weeks after quitting^a^: 37.8%; SEQ-Fluoxetine: 46.7% vs ST—Fluoxetine: 23.1% vs TNP: 41.2%, *p* = NA PP at 26 weeks after quitting^a^: 26.7%; SEQ—Fluoxetine: 40% vs ST—Fluoxetine: 15.4% vs TNP: 23.5%, *p* = NA	Among the total sample, participants in SEQ-Fluoxetine relative to ST-Fluoxetine, showed lower postquit depressive symptoms*, but not compared with the TNP group (*B* = ‒ 1.56; *SE* = 0.92; *Z* = 2.85; *p* = 0.092) Females reported greater postquitdepressive symptoms compared to men(*B* = 2.47; *SE* = 0.86; *Z* = 8.20; *p* = 0.004)
Muñoz et al. (1997)	PP at 3 months: inmediate condition: 22.5% vs delayed condition: 10.8%, *p* = .04 PP at 3 months: inmediate condition vs delayed condition: No MDEHx: 23.5% vs 7.7%, *p* = .12, current MDE: 14.3% vs 12%, *p* = .41, Hx MDE: 30.8% vs 11.1%, *p* = .04 PP at 6 months follow-up: inmediate condition 25.4% vs delayed condition: 9.2%, *p* = .01 PP at 6 months follow-up: inmediate condition vs delayed condition: No MDE Hx: 17.6% vs 15.4%, *p* = .49, current MDE: 17.9% vs 8%, *p* = .15, Hx MDE: 38.5% vs 7.4%, *p* = .01	No significant effects of treatment condition on CES-D scores at 3 months F (l,111) = 2.62, *p ≤*.109 CES-D at 3 months: inmediate condition vs delayed condition (*M* = 14 vs *M* = 16.7)
Muñoz et al. (2006)(study 3)	PP by depression diagnosis: PP at 1 month follow-up: No MDEHx: 16.4%, current MDE: 15.6%, Hx MDE: 18.9%, *p* ≥ .05 PP at 3 months follow-up: No MDEHx: 13.3%, current MDE: 3.1%, Hx MDE: 13.2%, *p* ≥ .05 PP at 6 months follow-up: No MDEHx: 12.8%, current MDE: 0%, Hx MDE: 15.1%, *p* ≥ .05 PP at 12 months follow-up: No MDEHx: 12.8%, current MDE: 9.4%, Hx MDE: 15.1%, *p* ≥ .05 PP by treatment condition:PP at 1 month follow-up: Guia+ITEMs: 17% vs Guía+ITEMs+MM: 16.5%, *p* ≥ .05 PP at 3 months follow-up: Guia+ITEMs: 12.1% vs Guía+ITEMs+MM: 12.2%, *p* ≥ .05 PP at 6 months follow-up: Guia+ITEMs: 13.52% vsGuía+ITEMs+MM:10.1%, *p* ≥ .05 PP at 12 months follow-up: Guia+ITEMs: 17% vs Guía+ITEMs+MM: 8.6%, *p* = .036	% of participants with depression diagnosis 1 month: 15.6 3 month: 3.1 6 month: 0 12 month: 9.4
Muñoz et al. (2006) (study 4)	PP by depression diagnosis: PP at 1 month follow-up: No MDEHx: 20.3%, current MDE: 14.6%, Hx MDE: 25%, *p* ≥ .05 PP at 3 months follow-up: No MDEHx: 21.4%, current MDE: 18.8%, Hx MDE: 38.5%, *p* = .025 PP at 6 months follow-up: No MDEHx: 21.9%, current MDE: 16.7%, Hx MDE: 34.6%, *p* = .05 PP at 12 months follow-up: No MDEHx: 19.3%, current MDE: 14.6%, Hx MDE: 36.5%, *p* = .012 PP by treatment condition: PP at 1 month follow-up: Guia+ITEMs: 23.3% vs Guía+ITEMs+MM: 16.9%, *p* ≥ .05 PP at 3 months follow-up: Guia+ITEMs: 28.1% vs Guía+ITEMs+MM: 19.7%, *p* ≥ .05 PP at 6 months follow-up: Guia+ITEMs: 26% vs Guía+ITEMs+MM: 20.4%, *p* ≥ .05 PP at 12 months follow-up: Guia+ITEMs: 22.6% vs Guía+ITEMs+MM: 20.4%, *p* ≥ .05	% of participants with depression diagnosis 1 month: 14.6 3 month: 18.8 6 month: 16.7 12 month: 14.6
Muñoz et al. (2009)	PP at 1 month: Guía: 17.4%, Guía+ITEMs: 19.1%, Guía+ITEMs+MM: 15.9%, Guía+ITEMs+MM+VG: 15.1%, *p* = NA PP at 1 month: current MDE vs. no MDE, p ≥ .05	Data not reported
	PP at 3 month: Guía: 16.6%, Guía+ITEMs: 17.9%, Guía+ITEMs+MM: 13.9%, Guía+ITEMs+MM+VG: 15.9%, *p* = NA PP at 6 month: Guía: 14.5%, Guía+ITEMs: 16.7%, Guía+ITEMs+MM: 14.3%, Guía+ITEMs+MM+VG: 12.7%, *p* = NA PP at 12 month: Guía: 19.8%, Guía+ITEMs: 19.1%, guía+ITEMs+MM: 20.7%, Guía+ITEMs+MM+VG: 22.7%, *p* = NA	
Patten et al. (2017)	PP at 12 week: Exercise: 73% vs Health education: 33%, *p* = .028 PP at 6 month: Exercise: 27% vs Health education: 40%, *p* = .48	PHQ9 at 12 week: Exercise vs Health education: (*M* = 7.4; *SD* = 4.5 vs *M* = 7.0; *SD* = 5.1)
Schnoll et al. (2010)	PP at 12 weeks: placebo vs bupropion: depressed: 7.4% vs 14.3%, *p* = NA, non-depressed: 28.6% vs 31.4%, *p* = NA PP at week 27: placebo vs bupropion: depressed: 7.4% vs 14.3%, *p* = NA, non-depressed: 20% vs 19.8%, *p* = NA	Data not reported
Thorsteinsson et al. (2001)	% of abstinents at day 29: NRT: 78% vs placebo: 50%, *p* ≤ .05	No significant effect of time on BDI scores Significant effect of time on HAM-D scores, %, *p* = .01 No significant interaction of time and smoking status on depression Active group had no effect on depressive symptoms
Van der Meer et al. (2010)	PA_b_ at 6 months: active: 30.5% vs control: 22.3%, *p* ≤ .05 PA at 12 months: active: 23.9% vs control: 14%, *p* ≤ .05 PP at 6 months: active: 37.4% vs control: 31%, *p* ≥ .05 PP at 12 months: active: 27.6% vs control: 24%, *p* ≥ .05	Quitting is associated with improvements in depressive symptoms among abstinents, especially from 0 to 6 months No intervention effect on depressive symptoms: Mean differences for depressive symptoms: 0–6 months: active: 1.1 vs control: 2.0 6–12 months: active: 0.6 vs control: 0.1
Vickers et al. (2009)	PP at EOT: exercise counseling: 17% vs health education condition: 23%, *p* = .75 PP at week 24: exercise counseling: 7% vs health education condition: 6.70%, *p* = 1.0	HAM-D at EOT: exercise counseling vs health education (*M* = 12.9; *SD* = 7.5 vs *M* = 12.0; *SD* = 7.8) HAM-D at week 24: exercise counseling vs health education (*M* = 7.4; *SD* = 4.6 vs *M* = 13.1; *SD* = 9.4)
Ward et al. (2013)	PA_c_ EOT: nicotine patch: 21.6% vs placebo 20%, *p* ≥ .05 6 months: nicotine patch: 13.4% vs placebo 14.1%, *p* ≥ .05 12 months: nicotine patch: 12.7% vs placebo 11.9%, *p* ≥ .05 PP EOT: nicotine patch: 25.4% vs placebo 25.9%, *p* ≥ .05 6 months: nicotine patch: 14.2% vs placebo 19.3%, *p* ≥ .05 12 months: nicotine patch: 20.1% vs placebo 14.8%, *p* ≥ .05	Data not reported

CA = Continuous abstinence; PP = Never smoking for 7 consecutive days; EOT = End of treatment; HADS-D = Hospital Anxiety and Depression Scale—Depression subscale; SDS = Medical Outcomes Survey Short Depression Screen; CBASP = Cognitive Behavioral Analysis System of Psychotherapy; HW = Health and Wellness; PA = Prolonged abstinence means that relapse is defined by smoking for 7 or more consecutive days or by smoking at least 1 cigarette over two consecutive weeks; Raw scores = Raw baseline scores on the CES-D (center for Epidemiological Studies Depression scale) within each quartile (e.g., I: sessions 1–4); NRT = Transdermal nicotine replacement therapy; CBT = Group cognitive behavioral therapy; UDD = Unipolar depressive symptoms; CD = Current depressive; PDO = past depression only; NHD = no history of depression; SEQ-Fluoxetine = Sequential fluoxetine treatment, starting 8 weeks since pre-quit; ST-Fluoxetine = Standard fluoxetine treatment; TNP = Transdermal nicotine patch; MDE = Major depressive episode; Hx = Lifetime, but not current MDE; ITEMs = Individually timed educational messages; MM = Mood management intervention; VG = Virtual group; PA_b_ = Prolonged abstinence is defined as not having smoked any cigarettes from month 2 to 6 and from month 2 to 12; PA_c_ = Prolonged abstinence is defined as complete abstinence after a two-week grace period following the quit day.

^a^Data provided by authors

The analyses of psychological treatments showed that trials that added a mood management component to a psychological smoking cessation intervention reported a mean point prevalence abstinence of 18.23% (ranging between 8.6% and 21.7%) at 6-month or longer follow-ups [23, 27–29]. While Cinciripini, Blalock [23] did not find significant treatment group differences in abstinence, Munoz, Lenert (28] (studies 3 and 4) reported lower abstinence rates among individuals assigned to a mood management intervention compared to those who were not. Similarly, Munoz, Barrera [27] did not report evidence of increased abstinence rates at 12 months among smokers allocated to a mood management intervention. Munoz, Marin [29] indicated that treating both smoking and depression simultaneously leads to higher abstinence rates compared to a delayed intervention, that is, treating abstinence at first followed by a mood management intervention after 3 months.

Hayes, Dunsiger [26] assessed the effect of a psychological treatment based on MI principles. Point prevalence and continuous abstinence rates at 12 months in the treatment condition were respectively 12.6% and 8.7%.

The analyses of effects of pharmacological treatments showed that the two studies using NRT as the sole pharmacotherapy improved abstinence rates compared to placebo. Concretely, Kinnunen, Korhonen (35] reported greater continuous abstinence rates at 12 months among patients assigned to nicotine gum (15.1%) than placebo (5.7%). Similarly, Thorsteinsson, Gillin (38] found a higher percentage of abstainers in the nicotine patch group (78%) compared to placebo (50%) during the acute phase over the first twenty-nine days.

Anthenelli, Morris (34] compared cessation outcomes among smokers assigned to varenicline or placebo and found 52-week point prevalence abstinence rates of 28.5% and 17.5%, respectively.

Ward, Asfar (39] assessed the comparative effectiveness of nicotine patch versus placebo. Considering point prevalence outcomes, a statistically significant effect for nicotine patch compared to placebo at both 6 (14.2% vs. 19.3%) and 12 months (20.1% vs. 18.4%) was found. Nonetheless, these differences faded when evaluating prolonged abstinence.

Two studies included antidepressants for smoking cessation. Minami, Kahler (36] compared the efficacy of three treatment conditions: sequential fluoxetine treatment, standard fluoxetine treatment and transdermal nicotine patch only. Results found point prevalence abstinence rates at 26 weeks after quitting, of 40% and 15.4% and 23.5%, respectively. No significant differences between treatment conditions were found. Schnoll, Martinez (37] showed that bupropion did not increase abstinence rates when compared to placebo among individuals receiving NRT either at 12 weeks or at week 27 (14.3% vs. 7.4%).

Seven studies investigated the effect of combined treatments for quitting. Evins, Culhane (40] indicated that bupropion did not increase the efficacy of receiving CBT and NRT. Smokers assigned to an exercise intervention combined with varenicline or NRT upon approval, did not show higher continuous abstinence rates (20%) than those assigned to a health education group (11.4%) [31]. Similarly, Vickers, Patten (32] did not report statistical significant differences in point prevalence abstinence at week 24 between individuals provided with exercise counseling (7%), and those receiving a health education intervention (6.7%). Patten, Bronars (33] found a positive effect of a supervised exercise intervention in enhancing abstinence rates at end-of-treatment (12 weeks) compared to a health education condition (73% vs 33%), but no statistically significant differences between groups were detected at 6-month follow-up.

Three studies included cognitive-behavioral cessation strategies as an adjunct to NRT. Catley, Ahluwalia (41] used culturally sensitive self-help material, including a guide and a video for depressed smokers. While these authors report abstinence rates at 6-month follow-up among the overall sample (25%), they do not account for treatment condition.

The second study examined the relationship between depression and smoking after receiving MI or CBT for quitting [25]. Again, although they report a 6-month follow-up abstinence of 21%, these authors do not account for treatment condition, so conclusions on the effect for each treatment cannot be established.

A third study mentioned above [32] evaluated the feasibility of an individualized exercise counseling intervention comprising cognitive behavioral strategies (e.g., discussion of benefits of exercise, positive reinforcement) for depressed smokers. Results did not indicate higher abstinence rates among individuals assigned to either group.

Trials that included a condition combining pharmacotherapy with a mood management intervention [24, 30–33] showed greater abstinence rates (about 21%) between 24 week and 12 or more follow-up sessions. Nonetheless, statistically significant differences were only found in van der Meer, Willemsen (30] (see Table 2).

Few studies analyze the differences in abstinence rates by depression status. None of them found statistically significant differences in smoking abstinence among depressed and non-depressed smokers [25, 27, 28, 35, 40].

### Systematic review: Depression outcomes

Twelve studies reported data on depression changes. Due to the heterogeneity of the data, a meta-analysis could not be performed (see Table 2). A significant effect of time on depression scores was reported by Thorsteinsson, Gillin (38] and Cinciripini, Blalock (23]. Most trials including depression-focused treatments reported an improvement in depression at the end of the intervention or in the long term [23, 28, 29, 31–33, 36, 40] (see Table 2). Nonetheless, statistically significant differences between treatment conditions and time were only found by Cinciripini, Blalock (23]. These authors concluded that depressed smokers receiving a psychological mood management treatment showed an amelioration of depressive symptoms compared to smokers receiving a health educational intervention. On the other hand, evidence was obtained regarding quitting and improvements on depressive symptoms, especially within the first 6 months after quitting [30, 40].

### Methodological quality ratings

Scoring in both individual and global ratings for each study included in the review is reported in Table 3. Overall, four (20%) of twenty studies were given a strong methodological rating. Seven studies (35%) were of moderate methodological quality and the remaining nine (45%) were scored as weak. Component ratings that reduced global quality ratings included lack of blinding in RCT designs, and high withdrawals and drop-outs rates at the final data collection.

**Table 3:** Methodological quality assessment.

	Selection bias	Study design	Confounders	Blinding	Data collection	Withdrawals	Global ratings
Anthenelli et al. (2003)	Strong	Strong	Strong	Strong	Strong	Moderate	Strong
Bernard et al. (2016)	Strong	Strong	Strong	Moderate	Strong	Weak	Moderate
Catley et al. (2003)	Weak	Strong	Weak	Moderate	Moderate	Moderate	Weak
Cinciripini et al. (2010)	Strong	Strong	Strong	Moderate	Strong	Strong	Strong
Evins et al. (2008)	Moderate	Strong	Strong	Strong	Strong	Weak	Moderate
Hall et al. (2006)	Weak	Strong	Weak	Moderate	Strong	Moderate	Weak
Hayes et al. (2010)	Weak	Strong	Weak	Moderate	Strong	Moderate	Weak
Japuntich et al. (2007)	Weak	Strong	Weak	Moderate	Strong	Weak	Weak
Kinnunen et al. (2008)	Moderate	Strong	Weak	Strong	Strong	Weak	Weak
Minami et al. (2015)	Moderate	Strong	Weak	Moderate	Moderate	Weak	Weak
Muñoz et al. (1997)	Moderate	Strong	Strong	Moderate	Strong	Weak	Moderate
Muñoz et al. (2006) (study 3)	Weak	Strong	Weak	Moderate	Moderate	Weak	Weak
Muñoz et al. (2006) (study 4)	Moderate	Strong	Weak	Moderate	Moderate	Moderate	Moderate
Muñoz et al. (2009)	Weak	Strong	Weak	Moderate	Moderate	Weak	Weak
Patten el al. (2017)	Moderate	Strong	Strong	Moderate	Strong	Strong	Strong
Schnoll et al. (2010)	Weak	Strong	Strong	Strong	Strong	Weak	Weak
Thorsteinsson et al. (2001)	Moderate	Strong	Weak	Strong	Strong	Moderate	Moderate
Van der Meer et al. (2010)	Strong	Strong	Strong	Moderate	Strong	Moderate	Strong
Vickers et al. (2009)	Moderate	Strong	Strong	Moderate	Strong	Weak	Moderate
Ward et al. (2013)	Moderate	Strong	Weak	Strong	Strong	Strong	Moderate

## Discussion

For the first time a systematic review and meta-analysis was conducted to summarize the evidence supporting the effectiveness of smoking cessation interventions among smokers solely with current depression. Previous reviews included patients who had a history of depression but not current depression or studies that did not assess the effects of the intervention and smoking status on depressive symptoms. When study findings were combined, the meta-analytic review revealed greater short-term and long-term smoking abstinence among intervention participants relative to participants in the control conditions. Subgroup analyses revealed stronger effects among studies that provided pharmacological treatments than in studies using psychological treatments.

Meta-analyses revealed that studies including psychological interventions showed a positive but not statistically significant effect on smoking cessation. These results should be interpreted with caution, since trials included had considerable variability in treatment type. Heterogeneity in protocols (e.g., treatment duration, face-to-face or self-help, amount of contact with a therapist) in similar types of treatment also prevents us from identifying clear and effective interventions.

Only one study evaluating a psychological mood management component as an adjunct to a smoking cessation intervention supported the inclusion of this component in smoking cessation interventions for people with current depression. Additionally, one study using BA reported significant intervention effects at 6- and 12-month follow-up. BA may serve to moderate the negative mood associated with quit attempts [7]. Nevertheless, it should be noted that none of the studies that included BA strategies incorporated a face-to-face BA treatment protocol, and that behavioral counseling delivered via written materials provided alongside other face-to-face interventions does not enhance cessation rates [43].

Consistent with a recent review [44], The three studies that examined the effect of exercise programmes on abstinence did not show significantly higher abstinence rates in the exercise group compared to a control condition at long-term follow-up. In the same line, the only study evaluating MI showed no significant changes in smoking abstinence at 12-month follow-up, suggesting that smokers with depression appear to require more intensive support to quit smoking.

Despite CBT being included in a number of studies, in most cases it is combined with pharmacotherapy (NRT or bupropion), so there is no available data on the differential effect of this approach on smoking behavior among people with current depression.

Surprisingly, no study has explored the effect of contingency management (CM), a well-established treatment for smoking [45–47].

Altogether, these results highlight the need to develop higher quality studies to strengthen the evidence based on the effectiveness of psychological treatments such as a mood management component, BA, CBT or CM.

Studies including pharmacological treatments showed a positive effect on smoking abstinence at short and at long-term follow-up. NRT and varenicline seem to increase smoking cessation compared to placebo which is in line with findings in the general population [48, 49]. Similarly, fluoxetine, taken in a sequential manner (8 weeks before quit day) appears to increase smoking abstinence in the long-term, although it does not have a clear advantage over nicotine patch treatment only. Nevertheless, the evidence of the effectiveness of these pharmacotherapies is very weak due to the small number of studies.

We found no evidence for the effectiveness of bupropion combined with CBT or NRT, although, again, there were only two trials investigating this drug in smokers with current depression.

Our findings showed no relationship between depression status and abstinence rates at follow-ups. Nevertheless, the evidence is insufficient since only five studies analyze the differences in abstinence rates by depression status.

An important finding of the current review was that most trials examining the impact of smoking cessation on depression reported an improvement in depressive symptoms. Smoking cessation in smokers with current depression does not appear to be associated with an increase in depressive symptoms and may actually lead to a reduced incidence of depression. Several factors such as the increase of self-efficacy and achievement associated with abstinence [38] and the effects of nicotine on dopamine [50] and the non-adrenalin receptor system [51] may have contributed to this result.

Taken together, these results suggest that healthcare providers should consider encouraging their patients with depression to seek smoking cessation services. Barriers to implementing smoking cessation interventions for patients with depression include limited knowledge of how to engage this population into treatment, and a belief that quitting may exacerbate depressive symptoms [15, 52, 53].

Gender differences in the association between smoking and depression have been scarcely studied. Consequently, studies analyzing the effectiveness of smoking cessation treatments for patients with current depression accounting for gender are needed.

Our systematic review and meta-analysis has some limitations. First, there was substantial heterogeneity in study design, which may impact comparability. One source of limited comparability is statistical adjustment for covariates. There was little consistency across studies regarding which covariates were included in models. A larger number of studies and increased standardization of analyses across studies would contribute to more precise meta-analytic results. Second, about 25% of reviewed studies did not confirm smoking cessation status with biochemical verification, so cessation outcomes reported in these trials may represent an overestimate. Third, we include four trials that have evaluated the effects of smoking cessation treatments in special populations of smokers, so the extent to which the results of these studies generalize to the general population or to other groups warrants further research. Fourth, nine studies received a methodological rating of weak. Fifth, although this review attempted to be as inclusive as possible, it was limited to studies that were published, studies that were available in the databases used, and studies including smoking and depression data. However, in studies that did not include sufficient data to confirm eligibility, we asked authors for additional data not supplied in the full text.

Strengths of this review include: the fact that it concentrated mostly on randomized controlled trials; the large sample in terms of the number of studies and number of participants across studies; and comparability of trials in the diagnosis of the participants (current depression) and in their definition and measurement of smoking abstinence and depression. Also, an established quality rating scale was used for data extraction and was completed independently by two researchers to minimize any rating errors. The broad search strategy used gives confidence that all currently available evidence has been identified in this review.

In conclusion, smoking cessation interventions, especially pharmacological treatments, appear to increase smoking abstinence in individuals with current depression. Nevertheless, there is insufficient evidence to draw strong conclusions regarding the effectiveness of any particular treatment model to optimally manage co-occurring smoking and depression due to the small number of studies. Moreover, most studies included in this review used designs that preclude us from yielding firm conclusions on both abstinence and depression outcomes. Heterogeneity in protocols in similar types of treatment also prevents us identifying clear effective interventions. However, our findings suggest some promising psychological and pharmacological smoking cessation strategies for patients with depression. Patients with depression can stop smoking and should be offered evidence-based smoking cessation treatments, including strategies that simultaneously target both depressive symptoms and smoking. Results also showed that smoking abstinence may be associated with an improvement in depressive symptoms. Future randomized clinical trials should be designed to test the effectiveness of smoking cessation treatments, relative to each other, for smokers with current depression and to analyze the key moderators that may influence treatment effectiveness.

## Supporting information

S1 Table PRISMA checklist. (DOC)

S1 Dataset Data underlying the meta-analysis. (XLSX)

## References

[1] World Health Organization. WHO Global Report. Mortality Attributable to Tobacco. Geneva: World Health Organization, 2012.

[2] Luger TM, Suls J, Vander Weg MW. How robust is the association between smoking and depression in adults? A meta-analysis using linear mixed-effects models. Addictive behaviors. 2014;39(10):1418–29. doi: 10.1016/j.addbeh.2014.05.011 . 24935795 10.1016/j.addbeh.2014.05.011

[3] Lasser K, Boyd JW, Woolhandler S, Himmelstein DU, McCormick D, Bor DH. Smoking and mental illness: A population-based prevalence study. Jama. 2000;284(20):2606–10. doi: 10.1001/jama.284.20.2606 . 11086367 10.1001/jama.284.20.2606

[4] Weinberger AH, Kashan RS, Shpigel DM, Esan H, Taha F, Lee CJ, et al Depression and cigarette smoking behavior: a critical review of population-based studies. The American journal of drug and alcohol abuse. 2016:1–16. doi: 10.3109/00952990.2016.1171327 . 27286288 10.3109/00952990.2016.1171327

[5] Breslau N, Peterson EL, Schultz LR, Chilcoat HD, Andreski P. Major depression and stages of smoking. A longitudinal investigation. Archives of general psychiatry. 1998;55(2):161–6. doi: 10.1001/archpsyc.55.2.161 9477930 10.1001/archpsyc.55.2.161

[6] Fluharty M, Taylor AE, Grabski M, Munafo MR. The Association of Cigarette Smoking With Depression and Anxiety: A Systematic Review. Nicotine & tobacco research: official journal of the Society for Research on Nicotine and Tobacco. 2017;19(1):3–13. doi: 10.1093/ntr/ntw140 . 27199385 10.1093/ntr/ntw140 PMC5157710

[7] Gierisch JM, Bastian LA, Calhoun PS, McDuffie JR, Williams JW Jr. Smoking cessation interventions for patients with depression: a systematic review and meta-analysis. J Gen Intern Med. 2012;27(3):351–60. doi: 10.1007/s11606-011-1915-2 . 22038468 10.1007/s11606-011-1915-2 PMC3286553

[8] van der Meer RM, Willemsen MC, Smit F, Cuijpers P. Smoking cessation interventions for smokers with current or past depression. Cochrane Database of Systematic Reviews. 2013; (8):CD006102 doi: 10.1002/14651858.CD006102.pub2 23963776 10.1002/14651858.CD006102.pub2 PMC13034420

[9] Secades-Villa R, Vallejo-Seco G, García-Rodríguez O, López-Núñez C, Weidberg S, González-Roz A. Contingency Management for Cigarette Smokers with Depressive Symptoms. Experimental and Clinical Psychopharmacology. 2015;23(5):351–60. doi: 10.1037/pha0000044 26280589 10.1037/pha0000044

[10] Blalock JA, Robinson JD, Wetter DW, Schreindorfer LS, Cinciripini PM. Nicotine withdrawal in smokers with current depressive disorders undergoing intensive smoking cessation treatment. Psychology of addictive behaviors: journal of the Society of Psychologists in Addictive Behaviors. 2008;22(1):122–8. doi: 10.1037/0893-164X.22.1.122 . 18298238 10.1037/0893-164X.22.1.122

[11] Mathew AR, Robinson JD, Norton PJ, Cinciripini PM, Brown RA, Blalock JA. Affective trajectories before and after a quit attempt among smokers with current depressive disorders. Nicotine & tobacco research: official journal of the Society for Research on Nicotine and Tobacco. 2013;15(11):1807–15. doi: 10.1093/ntr/ntt036 . 23509093 10.1093/ntr/ntt036 PMC3790623

[12] Hitsman B, Papandonatos GD, McChargue DE, DeMott A, Herrera MJ, Spring B, et al Past major depression and smoking cessation outcome: a systematic review and meta-analysis update. Addiction. 2013;108(2):294–306. doi: 10.1111/add.12009 . 23072580 10.1111/add.12009 PMC3593055

[13] Weinberger AH, Mazure CM, Morlett A, McKee SA. Two decades of smoking cessation treatment research on smokers with depression: 1990–2010. Nicotine & tobacco research: official journal of the Society for Research on Nicotine and Tobacco. 2013;15(6):1014–31. doi: 10.1093/ntr/nts213 . 23100459 10.1093/ntr/nts213 PMC3693502

[14] Taylor G, McNeill A, Girling A, Farley A, Lindson-Hawley N, Aveyard P. Change in mental health after smoking cessation: systematic review and meta-analysis. Bmj. 2014;348:g1151 doi: 10.1136/bmj.g1151 . 24524926 10.1136/bmj.g1151 PMC3923980

[15] Prochaska JJ. Failure to treat tobacco use in mental health and addiction treatment settings: a form of harm reduction? Drug and alcohol dependence. 2010;110(3):177–82. doi: 10.1016/j.drugalcdep.2010.03.002 . 20378281 10.1016/j.drugalcdep.2010.03.002 PMC2916693

[16] Moher D, Liberati A, Tetzlaff J, Altman DG, Group P. Preferred reporting items for systematic reviews and meta-analyses: the PRISMA statement. Annals of internal medicine. 2009;151(4):264–9, W64 . 19622511 10.7326/0003-4819-151-4-200908180-00135

[17] Borenstein M, Rothstein H. Comprehensive meta analysis: A computer program for research synthesis. Englewood, NJ: Biostat, Inc; 1999.

[18] Prochaska JJ, Delucchi K, Hall SM. A meta-analysis of smoking cessation interventions with individuals in substance abuse treatment or recovery. Journal of consulting and clinical psychology. 2004;72(6):1144–56. doi: 10.1037/0022-006X.72.6.1144 . 15612860 10.1037/0022-006X.72.6.1144

[19] Fleiss JL. Analysis of data from multiclinic trials. Controlled clinical trials. 1986;7(4):267–75. . 3802849 10.1016/0197-2456(86)90034-6

[20] Borenstein M, Hedges LV, Higgins JPT, Rothstein HR. Introduction to metaanalysis. Chichester, UK: Wiley; 2009.

[21] Armijo-Olivo S, Stiles CR, Hagen NA, Biondo PD, Cummings GG. Assessment of study quality for systematic reviews: a comparison of the Cochrane Collaboration Risk of Bias Tool and the Effective Public Health Practice Project Quality Assessment Tool: methodological research. Journal of evaluation in clinical practice. 2012;18(1):12–8. doi: 10.1111/j.1365-2753.2010.01516.x . 20698919 10.1111/j.1365-2753.2010.01516.x

[22] Deeks JJ, Dinnes J, D’Amico R, Sowden AJ, Sakarovitch C, Song F, et al Evaluating non-randomised intervention studies. Health technology assessment. 2003;7(27):iii–x, 1–173. . 14499048 10.3310/hta7270

[23] Cinciripini PM, Blalock JA, Minnix JA, Robinson JD, Brown VL, Lam C, et al Effects of an intensive depression-focused intervention for smoking cessation in pregnancy. Journal of consulting and clinical psychology. 2010;78(1):44–54. doi: 10.1037/a0018168 . 20099949 10.1037/a0018168 PMC2881321

[24] Hall SM, Tsoh JY, Prochaska JJ, Eisendrath S, Rossi JS, Redding CA, et al Treatment for cigarette smoking among depressed mental health outpatients: a randomized clinical trial. American journal of public health. 2006;96(10):1808–14. doi: 10.2105/AJPH.2005.080382 . 17008577 10.2105/AJPH.2005.080382 PMC1586139

[25] Japuntich SJ, Smith SS, Jorenby DE, Piper ME, Fiore MC, Baker TB. Depression predicts smoking early but not late in a quit attempt. Nicotine & tobacco research: official journal of the Society for Research on Nicotine and Tobacco. 2007;9(6):677–86. doi: 10.1080/14622200701365301 . 17558825 10.1080/14622200701365301

[26] Hayes RB, Dunsiger S, Borrelli B. The influence of quality of life and depressed mood on smoking cessation among medically ill smokers. Journal of behavioral medicine. 2010;33(3):209–18. doi: 10.1007/s10865-010-9254-z . 20204491 10.1007/s10865-010-9254-z

[27] Munoz RF, Barrera AZ, Delucchi K, Penilla C, Torres LD, Perez-Stable EJ. International Spanish/English Internet smoking cessation trial yields 20% abstinence rates at 1 year. Nicotine & tobacco research: official journal of the Society for Research on Nicotine and Tobacco. 2009;11(9):1025–34. doi: 10.1093/ntr/ntp090 . 19640833 10.1093/ntr/ntp090 PMC2725004

[28] Munoz RF, Lenert LL, Delucchi K, Stoddard J, Perez JE, Penilla C, et al Toward evidence-based Internet interventions: A Spanish/English Web site for international smoking cessation trials. Nicotine & tobacco research: official journal of the Society for Research on Nicotine and Tobacco. 2006;8(1):77–87. doi: 10.1080/14622200500431940 . 16497602 10.1080/14622200500431940

[29] Munoz RF, Marin BV, Posner SF, Perez-Stable EJ. Mood management mail intervention increases abstinence rates for Spanish-speaking Latino smokers. American journal of community psychology. 1997;25(3):325–43. . 9332966 10.1023/a:1024676626955

[30] van der Meer RM, Willemsen MC, Smit F, Cuijpers P, Schippers GM. Effectiveness of a mood management component as an adjunct to a telephone counselling smoking cessation intervention for smokers with a past major depression: a pragmatic randomized controlled trial. Addiction. 2010;105(11):1991–9. doi: 10.1111/j.1360-0443.2010.03057.x . 20735366 10.1111/j.1360-0443.2010.03057.x

[31] Bernard P, Ninot G, Cyprien F, Courtet P, Guillaume S, Georgescu V, et al Exercise and Counseling for Smoking Cessation in Smokers With Depressive Symptoms: A Randomized Controlled Pilot Trial. Journal of dual diagnosis. 2015;11(3–4):205–16. doi: 10.1080/15504263.2015.1113842 . 26683252 10.1080/15504263.2015.1113842

[32] Vickers KS, Patten CA, Lewis BA, Clark MM, Ussher M, Ebbert JO, et al Feasibility of an exercise counseling intervention for depressed women smokers. Nicotine & tobacco research: official journal of the Society for Research on Nicotine and Tobacco. 2009;11(8):985–95. doi: 10.1093/ntr/ntp101 . 19541948 10.1093/ntr/ntp101 PMC2711987

[33] Patten CA, Bronars CA, Vickers Douglas KS, Ussher MH, Levine JA, Tye SJ, et al Supervised, Vigorous Intensity Exercise Intervention for Depressed Female Smokers: A Pilot Study. Nicotine & tobacco research: official journal of the Society for Research on Nicotine and Tobacco. 2017;19(1):77–86. doi: 10.1093/ntr/ntw208 . 27613946 10.1093/ntr/ntw208 PMC5157716

[34] Anthenelli RM, Morris C, Ramey TS, Dubrava SJ, Tsilkos K, Russ C, et al Effects of varenicline on smoking cessation in adults with stably treated current or past major depression: a randomized trial. Annals of internal medicine. 2013;159(6):390–400. doi: 10.7326/0003-4819-159-6-201309170-00005 . 24042367 10.7326/0003-4819-159-6-201309170-00005

[35] Kinnunen T, Korhonen T, Garvey AJ. Role of nicotine gum and pretreatment depressive symptoms in smoking cessation: twelve-month results of a randomized placebo controlled trial. International journal of psychiatry in medicine. 2008;38(3):373–89. doi: 10.2190/PM.38.3.k . 19069579 10.2190/PM.38.3.k

[36] Minami H, Kahler C, Bloom E, Strong D, Abrantes A, Zywiak W, et al Effects of depression history and sex on the efficacy of sequential versus standard fluoxetine for smoking cessation in elevated depressive symptom smokers. Addictive Disorders & Their Treatment. 2015;14(1):29–39. doi: 10.1097/ADT.0000000000000042

[37] Schnoll RA, Martinez E, Tatum KL, Weber DM, Kuzla N, Glass M, et al A bupropion smoking cessation clinical trial for cancer patients. Cancer causes & control: CCC. 2010;21(6):811–20. doi: 10.1007/s10552-010-9507-8 . 20087643 10.1007/s10552-010-9507-8

[38] Thorsteinsson HS, Gillin JC, Patten CA, Golshan S, Sutton LD, Drummond S, et al The effects of transdermal nicotine therapy for smoking cessation on depressive symptoms in patients with major depression. Neuropsychopharmacology. 2001;24(4):350–8. doi: 10.1016/S0893-133X(00)00217-7 . 11182530 10.1016/S0893-133X(00)00217-7

[39] Ward KD, Asfar T, Al Ali R, Rastam S, Weg MW, Eissenberg T, et al Randomized trial of the effectiveness of combined behavioral/pharmacological smoking cessation treatment in Syrian primary care clinics. Addiction. 2013;108(2):394–403. doi: 10.1111/j.1360-0443.2012.04048.x . 22882805 10.1111/j.1360-0443.2012.04048.x PMC7942391

[40] Evins AE, Culhane MA, Alpert JE, Pava J, Liese BS, Farabaugh A, et al A controlled trial of bupropion added to nicotine patch and behavioral therapy for smoking cessation in adults with unipolar depressive disorders. Journal of clinical psychopharmacology. 2008;28(6):660–6. doi: 10.1097/JCP.0b013e31818ad7d6 . 19011435 10.1097/JCP.0b013e31818ad7d6 PMC3505846

[41] Catley D, Ahluwalia JS, Resnicow K, Nazir N. Depressive symptoms and smoking cessation among inner-city African Americans using the nicotine patch. Nicotine & tobacco research: official journal of the Society for Research on Nicotine and Tobacco. 2003;5(1):61–8. . 12745507

[42] McFall M, Saxon AJ, Malte CA, Chow B, Bailey S, Baker DG, et al Integrating tobacco cessation into mental health care for posttraumatic stress disorder: a randomized controlled trial. Jama. 2010;304(22):2485–93. doi: 10.1001/jama.2010.1769 . 21139110 10.1001/jama.2010.1769 PMC4218733

[43] Lancaster T, Stead LF. Self-help interventions for smoking cessation. The Cochrane database of systematic reviews. 2005; (3):CD001118 doi: 10.1002/14651858.CD001118.pub2 . 16034855 10.1002/14651858.CD001118.pub2

[44] Ussher MH, Taylor AH, Faulkner GE. Exercise interventions for smoking cessation. The Cochrane database of systematic reviews. 2014; (8):CD002295 doi: 10.1002/14651858.CD002295.pub5 . 25170798 10.1002/14651858.CD002295.pub5

[45] Ledgerwood DM. Contingency management for smoking cessation: where do we go from here? Curr Drug Abuse Rev. 2008;1(3):340–9. . 19630730 10.2174/1874473710801030340

[46] Sigmon SC, Patrick ME. The use of financial incentives in promoting smoking cessation. Prev Med. 2012;55 Suppl:S24–32. doi: 10.1016/j.ypmed.2012.04.007 . 22525802 10.1016/j.ypmed.2012.04.007 PMC3411852

[47] Cahill K, Hartmann-Boyce J, Perera R. Incentives for smoking cessation. The Cochrane database of systematic reviews. 2015; (5):CD004307 doi: 10.1002/14651858.CD004307.pub5 . 25983287 10.1002/14651858.CD004307.pub5

[48] Cahill K, Stevens S, Lancaster T. Pharmacological treatments for smoking cessation. Jama. 2014;311(2):193–4. doi: 10.1001/jama.2013.283787 . 24399558 10.1001/jama.2013.283787

[49] Mills EJ, Wu P, Lockhart I, Thorlund K, Puhan M, Ebbert JO. Comparisons of high-dose and combination nicotine replacement therapy, varenicline, and bupropion for smoking cessation: a systematic review and multiple treatment meta-analysis. Annals of medicine. 2012;44(6):588–97. doi: 10.3109/07853890.2012.705016 . 22860882 10.3109/07853890.2012.705016

[50] Fowler JS, Volkow ND, Wang GJ, Pappas N, Logan J, MacGregor R, et al Inhibition of monoamine oxidase B in the brains of smokers. Nature. 1996;379(6567):733–6. doi: 10.1038/379733a0 . 8602220 10.1038/379733a0

[51] Prochazka AV, Weaver MJ, Keller RT, Fryer GE, Licari PA, Lofaso D. A randomized trial of nortriptyline for smoking cessation. Arch Intern Med. 1998;158(18):2035–9. . 9778204 10.1001/archinte.158.18.2035

[52] Hall SM, Prochaska JJ. Treatment of smokers with co-occurring disorders: emphasis on integration in mental health and addiction treatment settings. Annual review of clinical psychology. 2009;5:409–31. doi: 10.1146/annurev.clinpsy.032408.153614 . 19327035 10.1146/annurev.clinpsy.032408.153614 PMC2718730

[53] Hitsman B, Moss TG, Montoya ID, George TP. Treatment of tobacco dependence in mental health and addictive disorders. Canadian journal of psychiatry Revue canadienne de psychiatrie. 2009;54(6):368–78. doi: 10.1177/070674370905400604 . 19527557 10.1177/070674370905400604 PMC3632078

